# Cancer awareness among adolescents in Britain: a cross-sectional study

**DOI:** 10.1186/1471-2458-12-580

**Published:** 2012-07-31

**Authors:** Richard G Kyle, Liz Forbat, Gill Hubbard

**Affiliations:** 1 Cancer Care Research Centre School of Nursing, Midwifery and Health, University of Stirling, Stirling, FK9 4LA, UK

## Abstract

**Background:**

Little is known about adolescents’ cancer awareness and help-seeking behaviour in Britain. This study assessed adolescents’: awareness of cancer symptoms, common cancers, and the relationship between cancer and age; anticipated delay and perceived barriers to seeking medical advice; and examined variation by age, gender, ethnicity and whether individuals knew someone with cancer.

**Methods:**

A survey was conducted using a modified paper version of the Cancer Awareness Measure (CAM). The sample included 478 adolescents (male: n = 250, 52.3%) aged 11–17 years old (mean = 13.8, SD = 1.24) recruited from four British schools between August and October 2011.

**Results:**

Adolescents’ cancer awareness was low. Half of all adolescents did not know the most common childhood (51%) or teenage (49%) cancers and most (69%) believed cancer was unrelated to age. Awareness of cancer symptoms was significantly higher among older adolescents (aged 13–17 years) (p = 0.003) and those who knew someone with cancer (p < 0.001). Three-quarters (74%) of adolescents indicated they would seek help for a symptom they thought might be cancer within 3 days, and half (48%) within 24 hours. The most endorsed barriers to help-seeking were ‘worry about what the doctor might find’ (72%), being ‘too embarrassed’ (56%), ‘too scared’ (54%) and ‘not feeling confident to talk about symptoms’ (53%). Endorsement of these emotional barriers was significantly higher among females (p ≤ 0.001).

**Conclusion:**

There are certain groups of adolescents with poor cancer awareness. Cancer messages need to be targeted and tailored to particular groups to prevent the emergence of health inequalities in adulthood. Interventions to raise adolescents’ cancer awareness have the potential for a life-long impact on encouraging early diagnosis and survival.

## Background

In the UK around 2,000 new diagnoses of cancer are made each year in teenagers and young adults (TYA) aged 15–24 years. This constitutes 0.6% of all cancer registrations. The most common cancer among males in the TYA age group is testicular cancer (27%), followed by Hodgkin Lymphoma (14%) and leukaemia (11%) and the most common cancers in young women are malignant melanoma (17%), Hodgkin Lymphoma (17%) and ovarian cancer (9%) [1].

In order to benchmark public awareness of cancer among British adults a population-based survey was conducted in 2008 using a validated instrument (Cancer Awareness Measure [CAM]). This study found that: recall was good for identifying the tumour symptom of lump/swelling (68%) but very poor for all other warning signs; the number of symptoms recognised in response to a closed question listing nine common cancer symptoms averaged seven out of nine; the most commonly endorsed barrier to seeking medical advice from a General Practitioner (GP) was a service barrier (difficulty making an appointment [41%]); and 82% correctly identified breast cancer as the most common in women and 43% correctly identified prostate cancer as the most common in men [2].

There are few studies that have reported adolescents’ cancer awareness. A recent study in England using the CAM found that 68% of adolescents (11–14 years old) stated that they did not know the most common cancer in teenagers and young adults or reported breast or lung cancer as the most common, despite these being rare among this age group [3]. A USA study of adolescents’ (11–18 years old) awareness of testicular cancer found that 73% of respondents had heard of it [4]; other studies indicate lower awareness (at 26% [5] and 47% [6]). A USA study of students’ (8–11 years old) knowledge about skin cancer found that less than 40% of adolescents answered questions correctly [7].

Self-examination for signs of cancers found in TYAs is also poor. A UK study suggests that the number of young males knowing how to, or who have performed, testicular self-examination is low [8]. One USA study (of 11–18 years) found that none of the male respondents knew how to perform testicular self-examination [9] and a Dutch study (15–19 years) found that only 2% regularly performed testicular self-examination [5]. A Turkish study found that 65% of female students (14–19 years) did not know how to breast self-examine [10].

Through the National Awareness and Early Diagnosis Initiative (NAEDI) in England and Detect Cancer Early (DCE) in Scotland, UK government health departments are committed to improving public cancer awareness, recognising it as one component of a comprehensive strategy to increase the proportion of people with early diagnosis [11,12]. This is because detecting cancer at an early stage reduces the risk of dying from the disease [13,14]. Late detection is multifactorial, but patient delay in visiting the GP may partially explain the problem [15-17] and lack of public awareness of cancer signs and symptoms may be further reasons for failing to make a GP appointment [18]. Nevertheless, reasons for not visiting the GP are complex and lack of awareness of signs and symptoms of cancer is just one of several determinant factors [19].

A study of adolescents’ cancer awareness was conducted in Scotland and England in 2011. The aims of the study were to: (1) address the relative lack of evidence of adolescents’ awareness of cancer; (2) assess the feasibility of using the CAM among an adolescent population; and (3) enable preliminary comparison between adolescents’ cancer awareness and benchmark data for adults’ cancer awareness. This paper reports adolescents’: awareness of cancer warning signs; anticipated delay; perceived barriers to seeking medical advice; knowledge of common childhood, teenage, male and female cancers; awareness of the relationship between cancer and age; and examines variation by gender, ethnicity, age and whether individuals reported that they knew someone with cancer.

## Methods

### Sample

To meet the aims of the study, data were collected from adolescents aged between 11 and 17 years old, recruited from four schools in Scotland and England between August and October 2011. Schools with an existing relationship with Teenage Cancer Trust were purposively sampled to maximise geographic and age distribution and to ensure both male and female adolescents were included in the study (i.e., single-sex schools were excluded). Thus, the sampling strategy incorporated elements of both convenience and purposive sampling.

### Survey instrument

Teachers administered a paper questionnaire to a whole class. Students were asked to complete the questionnaire in complete silence but were informed that it was not a test. Teachers encouraged students to complete as much of the questionnaire as they could. Students were allowed as much time as they needed within the 55 minute lesson to complete the questionnaire, although most did so within 20 minutes. The instrument incorporated the CAM and socio-demographic questions.

#### Cancer Awareness

Cancer awareness was assessed using items from the CAM, details of which are published elsewhere [20]. Before use of the CAM with an adolescent population a consultation was conducted with adolescents from Highland Youth Voice (a youth parliament for the Scottish Highlands [21]) to assess face validity of the instrument. This consultation was guided by principles and procedures of cognitive interviewing. Four Highland Youth Voice representatives participated in a three hour discussion with two researchers on a single afternoon in August 2011. Adolescents completed each question in turn individually while verbalising their thoughts as they completed the instrument. Group discussion followed to provide an initial indication of construct validity among adolescents. Participants were asked to reflect upon the meaning of their responses and whether their response had been shaped by their (mis)interpretation of individual questions. Researchers recorded in writing adolescents’ suggested changes to the instrument and their reasons for these changes. Participants recommended only two minor modifications to the CAM:

 1. Within the CAM respondents are asked to assess anticipated delay: ‘If you had a symptom that you thought might be a sign of cancer how soon would you contact your doctor to make an appointment to discuss it?’. Question wording remained unchanged from the CAM [19]. However, in this study the number of closed response options typically used in analysis and reporting of the CAM [2] were reduced from ten to six. Categories used in the CAM were: 1–3 days/4–6 days/1 week/2 weeks/1 month/6 weeks/3 months/6 months/12 months/never. Response options provided for this study were: within 24 hours/between 2 to 3 days/between 4 and 10 days/within a month/longer than a month/don’t know. This recommended change was made to simplify the questionnaire while retaining its ability to identify individuals who may delay help-seeking for longer than a month.

 2. Two additional questions concerning common childhood and TYA cancers were asked: ‘What do you think is the most common cancer in {children (12 years of age or younger)/teenagers and young adults (between 13 and 24 years of age)}?’. This recommended change was made to increase the relevance of the CAM to adolescents.

#### Socio-demographic characteristics

Socio-demographic questions were included to gather data on: age, gender, ethnicity (using census categories), and whether the student had been diagnosed with cancer or knew a relative or friend who had been diagnosed with cancer.

### Analysis

Data were analysed using SPSS 19.0. Descriptive statistics were calculated for demographic variables (i.e., age, gender, ethnicity, knowing someone with cancer), and CAM items. An independent samples *t*-test was used to examine differences in the mean number of cancer warning signs recognised by age, gender, ethnicity and knowing someone with cancer. Due to non-normal distributions a Mann–Whitney test was used to examine differences in the mean number of barriers to help-seeking endorsed by the same four demographic variables. Pearson’s chi-square (*χ*^2^) tests were used to examine relationships between CAM variables and dichotomised demographic variables (i.e., gender [Male/Female], ethnicity [White/Other ethnic background], age [younger (11–12 years)/older (13–17 years) adolescents], and knowing someone with cancer [Yes/No]). Ethnicity was dichotomised into White/Other ethnic background following established CAM reporting practice [2]. Age was dichotomised as interventions that seek to increase cancer awareness, which this study seeks to inform, may need to be attuned to this distinction. Analysis of covariance (ANCOVA) was used to identify independent predictors of recognition of cancer warning signs and endorsement of barriers to help-seeking. To enable ANCOVA of barriers endorsed these data were (square root) transformed to correct a positively skewed distribution. Thirty-seven adolescents (7.7%) did not wish to answer the question about whether they knew someone with cancer and an additional 19 (4.0%) were unclassified to age and/or ethnicity. Fifty-six adolescents (11.7%) were therefore excluded from ANCOVA that included these independent variables.

### Ethical considerations

Approval for the study was obtained from the Research Ethics Committee in the School of Nursing, Midwifery and Health, University of Stirling. Parents/carers were informed of the study by letter and could opt their child out of the research, although none chose to do so. Written informed consent was obtained from each adolescent before completion of the questionnaire.

## Results

### Sample

The sample included 478 adolescents (male: n = 250, 52.3%) aged between 11 and 17 years old (mean age = 13.8, Standard Deviation [SD] = 1.24). Socio-demographic characteristics of respondents are shown in Table 1.

**Table 1:** Sample demographic characteristics

	**n**	**%**
*Gender*		
Male	250	52.3
Female	228	47.7
*Age*^*†*^		
11	1	0.2
12	107	23.4
13	25	5.5
14	193	42.1
15	99	21.6
16	29	6.3
17	4	0.9
*Ethnicity*^*‡*^		
White	438	93.2
Other ethnic backgrounds	32	6.8
* Mixed*	*17*	*3.6*
* Asian*	*7*	*1.5*
* Black*	*2*	*0.4*
* Other*	*6*	*1.3*
*Knew someone with cancer*^*§*^		
Yes	292	66.2
No	149	33.8
*Region*		
Scottish Highlands	155	32.4
South West England	156	32.6
English East Midlands	29	6.1
North West England	138	28.9
*Country*		
Scotland	155	32.4
England	323	67.6

Notes:

^†^ Age not reported by 20 adolescents.

^‡^ Ethnicity not reported by 8 adolescents.

^§^ Thirty-seven adolescents did not wish to answer this question.

### Recall of cancer warning signs

Recall of cancer warning signs among young people was poor. One in four adolescents (26.2%) did not know a sign or symptom of cancer. ‘Lump or swelling’ showed the highest level of recall (64.4%) but recall of other cancer warning signs was very poor (e.g., 13.8% for both ‘unexplained pain’ and a ‘change in the appearance of a mole’, 6.5% for ‘weight loss’ and 1.3% for ‘a sore that does not heal’). Misconceptions were also evident with around one in seven (14.4%) young people reporting ‘hair loss’ as a cancer warning sign and this was the second most frequently recalled symptom. Table 2 shows cancer warning signs recalled.

**Table 2:** Recall of cancer warning signs

	**Yes**	
	**n**	**%**
Lump or swelling	308	64.4
Did not know	125	26.2
Hair loss	69	14.4
Pain	66	13.8
Change in appearance of mole	66	13.8
Nausea/Sickness	45	9.4
Headache/Migraine	45	9.4
Bleeding	40	8.4
Cough	39	8.2
Tiredness/Fatigue	33	6.9
Generally unwell	32	6.7
Weight loss	31	6.5
Tumour/Growth	17	3.6
Breathing problems	16	3.3
Spots/rashes	14	2.9
Feeling weak	10	2.1
Bruising	8	1.7
Bowel/Bladder Habits	7	1.5
Dizziness	7	1.5
Sore that doesn't heal	6	1.3
Blurred vision	5	1.0
Flu symptoms	5	1.0
Swallowing	4	0.8
Stomach ache	4	0.8
Sore throat	4	0.8
Loss of appetite	3	0.6
Cramps	3	0.6
Infection	3	0.6
Weight gain	2	0.4

The percentage of boys stating that they did not know a warning sign or symptom was almost twice that of girls (34.0% vs. 17.5%, *χ*^2^(1, 478) = 16.72, p < 0.001) and the percentage of participants from other ethnic backgrounds who could not recall at least one symptom was 1.75 times that of white adolescents (43.8% vs. 24.9%, *χ*^2^(1, 470) = 5.49, p = 0.019). The percentage of those reporting that they did not know someone with cancer who could not recall a sign or symptom of cancer was nearly twice that of those who did know someone with cancer (36.2% vs. 18.8%, *χ*^2^(1, 441) = 16.06, p < 0.001).

### Recognition of cancer warning signs

‘Lump or swelling’ was the most recognised symptom (89.3%) followed by ‘change in the appearance of a mole’ (58.8%) and ‘persistent change in bowel/bladder habits’ (53.6%). Around half recognised ‘unexplained bleeding’ (48.5%) or ‘persistent unexplained pain’ (45.8%) as cancer warning signs, two in five recognised ‘unexplained weight loss’ (40.6%) and around one in three recognised ‘difficulty swallowing’ (35.6%) or a ‘persistent cough or hoarseness’ (31.8%). The least recognised symptom (‘a sore that does not heal’) was recognised by one in four adolescents (23.8%).

Knowing someone with cancer resulted in higher recognition of all nine cancer warning signs and these relationships were significant for four symptoms: ‘change in bowel or bladder habits’ (*χ*^2^(1, 438) = 13.44, p < 0.001); ‘unexplained bleeding’ (*χ*^2^(1, 439) = 10.64, p = 0.001); ‘change in the appearance of a mole’ (*χ*^2^(1, 437) = 11.36, p = 0.001); and ‘lump or swelling’ (*χ*^2^(1, 439) = 6.20, p = 0.013) (Table 3). With the exception of ‘change in bowel or bladder habits’, more older (13–17 years) than younger (11–12 years) adolescents recognised each of the nine cancer warning signs and there was a significant association between age and recognition for three symptoms: ‘lump or swelling’ (*χ*^2^(1, 456) = 17.02, p < 0.001); ‘difficulty swallowing’ (*χ*^2^(1, 454) = 4.82, p = 0.028); and ‘cough or hoarseness’ (*χ*^2^(1, 456) = 4.66, p = 0.031) (Table 3).

**Table 3:** Recognition of cancer warning signs by gender, ethnicity, age and knowing someone with cancer

**Cancer warning sign % Yes (n)**	**Gender (n = 478)**			**Ethnicity (n = 470)**			**Age (n = 458)**			**Knew someone with cancer (n = 441)**		
	**Male**	**Female**	Significance*	**White**	**Other**	Significance*	**Child**	**Teenager**	Significance*	**Yes**	**No**	Significance*
	**(n = 250)**	**(n = 228)**		**(n = 438)**	**(n = 32)**		**(n = 108)**	**(n = 350)**		**(n = 292)**	**(n = 149)**	
Lump or swelling	88.3 (219)	91.2 (208)	χ^2^(1, 476) = 1.10	89.7 (391)	90.6 (29)	χ^2^(1, 468) = 0.03	**80.4 (86)**	**93.7 (327)**	**χ**^**2**^**(1, 456) = 17.02**	**93.1 (271)**	**85.8 (127)**	**χ**^**2**^**(1, 439) = 6.20**
			p = 0.295			p = 1.000†			**p < 0.001**			**p = 0.013**
Unexplained pain	47.6 (118)	44.7 (101)	χ^2^(1, 474) = 0.40	46.0 (200)	45.2 (14)	χ^2^(1, 466) = 0.01	38.3 (41)	48.1 (167)	χ^2^(1, 454) = 3.17	48.1 (139)	41.9 (62)	χ^2^(1, 437) = 1.52
			p = 0.528			p = 0.930			p = 0.075			p = 0.218
Unexplained bleeding	48.0 (119)	49.6 (113)	χ^2^(1, 476) = 0.12	49.2 (215)	41.9 (13)	χ^2^(1, 468) = 0.61	43.5 (47)	50.6 (176)	χ^2^(1, 456) = 1.64	**54.3 (158)**	**37.8 (56)**	**χ**^**2**^**(1, 439) = 10.64**
			p = 0.731			p = 0.434			p = 0.200			**p = 0.001**
Cough or hoarseness	**36.7 (91)**	**26.8 (61)**	**χ**^**2**^**(1, 476) = 5.40**	31.8 (139)	25.8 (8)	χ^2^(1, 468) = 0.48	**23.1 (25)**	**34.2 (119)**	**χ**^**2**^**(1, 456) = 4.66**	35.4 (103)	28.4 (42)	χ^2^(1, 439) = 2.18
			**p = 0.020**			p = 0.487			**p = 0.031**			p = 0.139
Change in bowel/bladder habits	54.4 (135)	53.3 (121)	χ^2^(1, 475) = 0.06	**55.5 (242)**	**35.5 (11)**	**χ**^**2**^**(1, 467) = 4.67**	56.5 (61)	53.9 (187)	χ^2^(1, 455) = 0.22	**60.3 (175)**	**41.9 (62)**	**χ**^**2**^**(1, 438) = 13.44**
			p = 0.805			**p = 0.031**			p = 0.637			**p < 0.001**
Difficulty swallowing	36.6 (90)	35.2 (80)	χ^2^(1, 473) = 0.09	35.4 (154)	41.9 (13)	χ^2^(1, 466) = 0.54	**26.9 (29)**	**38.4 (133)**	**χ**^**2**^**(1, 454) = 4.82**	36.2 (105)	33.6 (49)	χ^2^(1, 436) = 0.30
			p = 0.761			p = 0.464			**p = 0.028**			p = 0.585
Change in appearance of a mole	**54.5 (134)**	**64.5 (147)**	**χ**^**2**^**(1, 474) = 4.90**	**60.8 (265)**	**41.9 (13)**	**χ**^**2**^**(1, 467) = 4.27**	51.9 (56)	62.5 (217)	χ^2^(1, 455) = 3.92	**66.7 (194)**	**50.0 (73)**	**χ**^**2**^**(1, 437) = 11.36**
			**p = 0.027**			**p = 0.039**			p = 0.048			**p = 0.001**
Sore that does not heal	**28.0 (69)**	**19.7 (45)**	**χ**^**2**^**(1, 474) = 4.48**	24.5 (107)	22.6 (7)	χ^2^(1, 467) = 0.06	20.4 (22)	25.6 (89)	χ^2^(1, 455) = 1.24	25.9 (75)	22.4 (33)	χ^2^(1, 437) = 0.61
			**p = 0.034**			p = 0.806			p = 0.265			p = 0.435
Unexplained weight loss	42.1 (104)	39.5 (90)	χ^2^(1, 475) = 0.34	41.3 (180)	32.3 (10)	χ^2^(1, 467) = 0.98	36.1 (39)	42.4 (147)	χ^2^(1, 455) = 1.33	43.8 (127)	35.8 (53)	χ^2^(1, 438) = 2.58
			p = 0.560			p = 0.323			p = 0.248			p = 0.108

Notes:

* Pearson’s *χ*^2^ test for 2×2 tables (i.e., Yes vs. No/Don’t know for each demographic variable). Statistically significant associations at the p < 0.05 level are emboldened.

† Fisher’s Exact Probability reported due to violation of *χ*^2^ assumption that tables should not have more than 20% of cells with an expected frequency of less than five.

The mean number of cancer warning signs recognised was 4.28 (SD = 2.14) out of 9. Boys recognised slightly more warning signs than girls (4.32, SD = 2.26 vs. 4.24, SD = 2.01), although this difference was not statistically significant (t(475.7) = 0.41, p = 0.685). White adolescents recognised more warning signs than those from other ethnic backgrounds (4.32, SD = 2.13 vs. 3.69, SD = 2.29) but this was also not significant (t(468) = 1.62, p = 0.106). There was a statistically significant difference in recognition between younger (11–12 years) and older (13–17 years) adolescents (3.76, SD = 2.05 vs. 4.46, SD = 2.13; t(456) = −3.03, p = 0.003). Adolescents who knew someone with cancer had significantly higher recognition than those who did not (4.61, SD = 1.94 vs. 3.74, SD = 2.34; t(439) = −3.95, p < 0.001).

In an ANCOVA of the total number of cancer warning signs recognised, being an older (13–17 years) adolescent (F(1, 417) = 7.35, p = 0.007) and knowing someone with cancer (F(1, 417) = 18.50, p < 0.001) were significant independent predictors.

### Awareness of relationship between cancer and age

The majority of adolescents believed that cancer was unrelated to age (68.5%). A statistically significantly higher percentage of female adolescents, white adolescents, and those who knew someone with cancer, believed cancer was unrelated to age (Gender: 79.9% vs. 57.4%, *χ*^2^(1, 454) = 26.67, p < 0.001; Ethnicity: 70.4% vs. 43.3%, *χ*^2^(1, 446) = 9.54, p = 0.002; Knew someone with cancer: 75.5% vs. 57.0%, *χ*^2^(1, 420) = 15.11, p < 0.001). Among adolescents who did not hold this belief, someone in their 20s was thought most likely to develop cancer in the next year (5.7%), followed by someone in their 80s (4.8%), and 30s (4.6%). Boys thought someone in their 20s was most likely to develop cancer in the next year (7.8%) followed by someone in their 80s (7.0%). Girls believed someone in their 30s was most likely (4.9%), followed by someone in their 20s (3.6%).

### Awareness of common cancers

**Figure 1:**
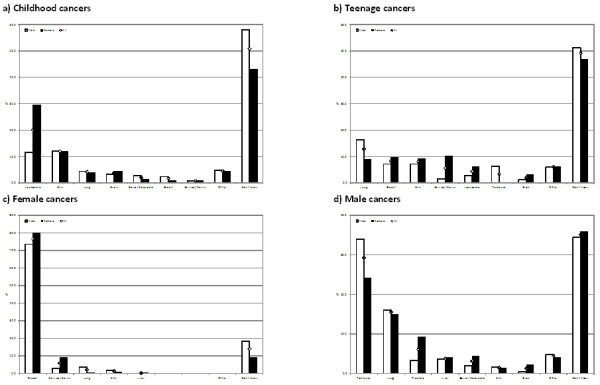
**Perceptions of childhood, teenage and female/male cancers by gender. a**) Childhood cancers **b**) Teenage cancers **c**) Female cancers **d**) Male cancers.

Half of adolescents reported that they did not know the most common childhood (50.8%) and teenage (49.2%) cancer (Figure 1a and 1b). One in five adolescents (20.1%) correctly identified leukaemia as the most common childhood cancer (Figure 1a). Lung cancer was considered the most common teenage cancer and was identified by 1 in 8 adolescents (12.8%) (Figure 1b). Three-quarters of adolescents (76.6%) correctly identified breast cancer as the most common female cancer. However, 1 in 7 (14.0%) reported that they did not know the most common cancer in women (Figure 1c). A third of adolescents (35.1%) did not know the most common male cancer and just under a third (29.3%) identified testicular cancer as the most common (Figure 1d).

A significantly higher percentage of boys compared to girls reported that they did not know the most common childhood (*χ*^2^(1, 478) = 10.76, p = 0.001) and female cancers (*χ*^2^(1, 478) = 8.36, p = 0.004) (Figure 1). A significantly higher percentage of adolescents who did not know someone with cancer, compared to those who did, reported that they did not know the most common cancer in children (*χ*^2^(1, 441) = 11.27, p = 0.001), teenagers (*χ*^2^(1, 441) = 9.17, p = 0.002), women (*χ*^2^(1, 441) = 10.39, p = 0.001) and men (*χ*^2^(1, 441) = 16.86, p < 0.001). A significantly higher percentage of adolescents from other ethnic backgrounds, compared to their White counterparts, reported that they did not know the most common childhood (*χ*^2^(1, 470) = 4.40, p = 0.036) and female cancers (*χ*^2^(1, 470) = 6.14, p = 0.013). Not knowing the most common male cancer was the only significant association with age: (*χ*^2^(1, 458) = 24.73, p < 0.001).

### Anticipated delay

Almost three-quarters of adolescents (73.5%) indicated that they would seek medical help for a symptom they thought might be cancer within three days, and around half (47.5%) would seek help within 24 hours. However, 1 in 10 adolescents (10.8%) responded that they did not know how long they would wait, and this was higher among boys (13.8% vs. Girls: 7.5%), adolescents from other ethnic backgrounds (16.1% vs. White: 10.2%), older (13–17 years) adolescents (11.0% vs. younger (11–12 years): 9.3%) and those who did not know someone with cancer (15.8% vs. Yes: 6.9%). A higher percentage of boys (28.9% vs. Girls: 23.9%), adolescents from other ethnic backgrounds (38.7% vs. White: 25.6%), older (13–17 years) adolescents (27.5% vs. younger (11–12 years): 24.1%) and those who did not know someone with cancer (29.5% vs. 24.5%) would delay longer than three days before seeking help and this pattern was also evident for anticipated delay >10 days. However, there were no statistically significant relationships between anticipated delay of >3 days or >10 days and gender, ethnicity, age or knowing someone with cancer.

### Barriers to help-seeking

**Figure 2:**
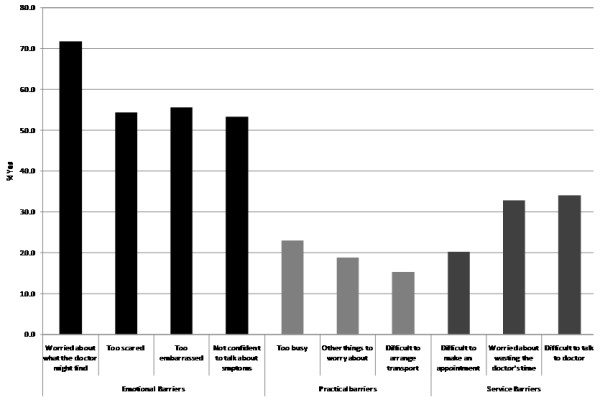
Barriers to help-seeking endorsed.

Emotional barriers were the most frequently endorsed, followed by service and practical barriers. ‘Worry about what the doctor might find’ was the most endorsed barrier (71.8%) and over half of adolescents reported being ‘too embarrassed’ (55.6%), ‘too scared’ (54.4%) or ‘not feeling confident to talk about symptoms’ (53.3%). Around a third of adolescents said service barriers including ‘difficulty talking to the doctor’ (34.1%) or ‘worry about wasting their time’ (32.8%) would put them off going to see the doctor. Practical barriers were least widely endorsed, although being ‘too busy’ was reported by around 1 in 4 adolescents (23.0%) and the least frequently endorsed barrier – ‘difficulty arranging transport’ – was a barrier for 1 in 7 adolescents (15.3%). Figure 2 shows the percentage of adolescents who endorsed each barrier.

There was evidence of significant associations between gender and endorsement of all four emotional barriers: ‘Worry about what the doctor might find’ (*χ*^2^(1, 476) = 22.95, p < 0.001); ‘too scared’ (*χ*^2^(1, 475) = 25.38, p < 0.001); ‘too embarrassed’ (*χ*^2^(1, 474) = 21.76, p < 0.001); ‘not confident to talk about symptoms’ (*χ*^2^(1, 475) = 10.60, p = 0.001). Only one other (service) barrier was significantly related to gender: ‘worry about wasting the doctor’s time’ (*χ*^2^(1, 474) = 8.00, p = 0.005). For each of these five barriers girls had a higher level of endorsement than boys (Table 4). Only ‘difficulty arranging transport’ and ‘being too busy’, respectively, were significantly related with ethnicity (*χ*^2^(1, 467) = 7.41, p = 0.016) and age (*χ*^2^(1, 454) = 5.26, p = 0.022). Adolescents from other ethnic backgrounds reported higher levels of endorsement with ‘difficulty arranging transport’ than white respondents, and teenagers reported being ‘too busy’ more often than children (Table 4). Knowing someone with cancer was only significantly related to the emotional barrier being ‘too scared’ (*χ*^2^(1, 438) = 4.42, p = 0.035).

**Table 4:** Barriers to help seeking by gender, ethnicity, age and knowing someone with cancer

**Barrier % Yes (n)**	**Gender (n = 478)**			**Ethnicity (n = 470)**			**Age (n = 458)**			**Knew someone with cancer (n = 441)**		
	**Male**	**Female**	Significance*	**White**	**Other**	Significance*	**Child**	**Teenager**	Significance*	**Yes**	**No**	Significance*
	**(n = 250)**	**(n = 228)**		**(n = 438)**	**(n = 32)**		**(n = 108)**	**(n = 350)**		**(n = 292)**	**(n = 149)**	
**Emotional Barriers**												
Worried about what the doctor might find	**62.7 (156)**	**82.4 (187)**	**χ**^**2**^**(1, 476) = 22.95**	72.5 (316)	62.5 (20)	χ^2^(1, 468) = 1.47	75.9 (82)	71.3 (248)	χ^2^(1, 456) = 0.90	74.7 (218)	68.7 (101)	χ^2^(1, 439) = 1.74
			**p < 0.001**			p = 0.226			p = 0.344			p = 0.187
Too scared	**43.8 (109)**	**66.8 (151)**	**χ**^**2**^**(1, 475) = 25.38**	55.2 (240)	50.0 (16)	χ^2^(1, 467) = 0.32	46.7 (50)	57.8 (201)	χ^2^(1, 455) = 4.03	**57.9 (169)**	**47.3 (69)**	**χ**^**2**^**(1, 438) = 4.42**
			**p < 0.001**			p = 0.570			p = 0.045			**p = 0.035**
Too embarrassed	**46.0 (114)**	**67.3 (152)**	**χ**^**2**^**(1, 474) = 21.76**	56.7 (246)	50.0 (16)	χ^2^(1, 466) = 0.54	49.5 (53)	58.5 (203)	χ^2^(1,454) = 2.68	58.1 (169)	50.0 (73)	χ^2^(1, 437) = 2.57
			**p < 0.001**			p = 0.462			p = 0.102			p = 0.109
Not confident to talk about symptoms	**46.6 (116)**	**61.5 (139)**	**χ**^**2**^**(1, 475) = 10.60**	54.7 (238)	40.6 (13)	χ^2^(1, 467) = 2.38	54.2 (58)	54.3 (189)	χ^2^(1, 455) < 0.01	54.1 (158)	50.7 (74)	χ^2^(1, 438) = 0.46
			**p = 0.001**			p = 0.123			p = 0.985			p = 0.498
**Practical Barriers**												
Too busy	23.0 (57)	23.5 (53)	χ^2^(1, 474) = 0.01	22.4 (97)	28.1 (9)	χ^2^(1,466) = 0.57	**15.0 (16)**	**25.6 (89)**	**χ**^**2**^**(1, 454) = 5.26**	21.2 (62)	23.4 (34)	χ^2^(1, 437) = 0.28
			p = 0.904			p = 0.452			**p = 0.022**			p = 0.598
Other things to worry about	17.0 (42)	21.2 (48)	χ^2^(1, 473) = 1.37	18.7 (81)	19.4 (6)	χ^2^(1, 465) = 0.01	13.1 (14)	21.1 (73)	χ^2^(1, 453) = 3.38	18.3 (53)	17.8 (26)	χ^2^(1, 436) = 0.01
			p = 0.241			p = 0.924			p = 0.066			p = 0.905
Difficult to arrange transport	16.5 (41)	14.2 (32)	χ^2^(1, 475) = 0.49	**13.6 (59)**	**31.3 (10)**	**χ**^**2**^**(1, 467) = 7.41**	16.8 (18)	14.9 (52)	χ^2^(1, 455) = 0.22	13.4 (39)	17.1 (25)	χ^2^(1, 438) = 1.11
			p = 0.486			**p = 0.016**†			p = 0.637			p = 0.293
**Service Barriers**												
Difficult to make an appointment	19.8 (49)	21.2 (48)	χ^2^(1, 473) = 0.14	19.4 (84)	31.3 (10)	χ^2^(1, 465) = 2.60	15.9 (17)	21.6 (75)	χ^2^(1, 454) = 1.66	20.3 (59)	17.8 (26)	χ^2^(1, 436) = 0.40
			p = 0.706			p = 0.107			p = 0.198			p = 0.528
Worried about wasting the doctor’s time	**27.3 (68)**	**39.6 (89)**	**χ**^**2**^**(1, 474) = 8.00**	33.8 (147)	28.1 (9)	χ^2^(1, 467) = 0.43	33.6 (36)	33.9 (118)	χ^2^(1, 455) < 0.01	32.9 (96)	31.0 (45)	χ^2^(1, 437) = 0.15
			**p = 0.005**			p = 0.512			p = 0.960			p = 0.698
Difficult to talk to doctor	30.6 (76)	38.5 (87)	χ^2^(1, 474) = 3.23	34.1 (148)	34.4 (11)	χ^2^(1, 466) < 0.01	37.4 (40)	33.4 (116)	χ^2^(1, 454) = 0.57	34.4 (100)	29.5 (43)	χ^2^(1, 437) = 1.07
			p = 0.072			p = 0.975			p = 0.452			p = 0.302

Notes:

* Pearson’s *χ*^2^ test for 2×2 tables (i.e., Yes vs. No/Don’t know for each demographic variable). Statistically significant associations at the p < 0.05 level are emboldened.

† Fisher’s Exact Probability reported due to violation of *χ*^2^ assumption that tables should not have more than 20% of cells with an expected frequency of less than five.

Adolescents endorsed an average of 3.79 (SD = 2.25) barriers out of 10. Girls endorsed significantly more barriers than boys (4.32, SD = 2.10 vs. 3.31, SD = 2.28; Mann–Whitney U = 20,751, p < 0.001). The mean number of barriers endorsed by white adolescents was slightly higher than for those from other ethnic backgrounds (3.78, SD = 2.23 vs. 3.75, SD = 2.59) and slightly higher among older (13–17 years) than younger (11–12 years) adolescents (3.90, SD = 2.31 vs. 3.56, SD = 2.10), although these differences were not significant (Ethnicity: Mann–Whitney U = 6,842, p = 0.821; Age: Mann–Whitney U = 17,373, p = 0.201). Adolescents who knew someone with cancer endorsed significantly more barriers than those who did not (3.85, SD = 2.15 vs. 3.46, SD = 2.27; Mann–Whitney U = 19,232.5, p = 0.044).

In an ANCOVA of the total number of barriers endorsed, being female was the only significant independent predictor (F(1, 417) = 24.77, p < 0.001).

## Discussion

### Cancer awareness

This study adds to a small but growing body of research around adolescents’ cancer awareness and confirms findings from studies conducted elsewhere that cancer awareness is low among this age group [4-7]. Moreover, it shows that adolescents in this study had lower cancer awareness than young (18–24 years old) and older British adults (25-65+ years old) [2,22]. For example, although recall of ‘lump or swelling’ as a cancer warning sign among adolescents was comparable to adults (64.4% vs. 67.9%), recall of ‘unexplained pain’ and ‘change in the appearance of a mole’ were lower among adolescents than adults (13.8% vs. 27.4% and 13.8% vs. 26.3%, respectively), and there was considerable disparity between adolescents’ and adults’ recall of ‘weight loss’ (6.5% vs. 26.7%) [2]. Moreover, on average, adolescents in this study recognised three fewer cancer warning signs than the adult population (4 vs. 7) [2]. Recognition of all nine cancer symptoms was lower among adolescents than adults and the greatest disparity was evident for ‘unexplained weight loss’ (40.6% vs. 83.2%), followed by ‘difficulty swallowing’ (35.6% vs. 76.9%) and ‘a sore that does not heal’ (23.8% vs. 61.4%) [2].

To inform strategies to improve early diagnosis it is important to understand how patterns of cancer awareness and help-seeking behaviour shift and solidify across the lifecourse. Adolescent health behaviours, such as help-seeking, track into adulthood and partially explain health inequalities in later life [23]. Differences in levels of cancer awareness and help-seeking between social groups that appear in adolescence, if not adequately addressed through early intervention, can become more established in adulthood.

Among British adults there were statistically significant differences in the mean number of cancer warning signs recognised by gender and ethnicity [22]. This study showed that these differences were also apparent among adolescents but were not statistically significant. This suggests there is an opportunity to intervene with adolescents so that differences in cancer awareness between gender and ethnic groups do not widen. Nevertheless, a larger nationally representative study of adolescents is required to verify these findings.

Two-thirds (66.2%) of the adolescents in this study knew someone with cancer. Knowing someone with cancer was associated with higher levels of recognition of all nine cancer warning signs. Adolescents who knew someone with cancer were also significantly more likely to be able to recall a cancer warning sign and less likely to report that they did not know the most common cancer in children, teenagers, women and men. Adolescents’ experiential knowledge of cancer is important in shaping their understanding of symptoms. There are health promotion opportunities therefore for increasing awareness of cancer in this age group, for example, through peer education.

### Barriers to help seeking

To inform strategies for encouraging prompt presentation it is important to understand reasons for patients delaying seeking help. Clinically useful models have been developed which identify potential areas of delay [15,17]. Although there is extensive literature on reasons for delays in cancer diagnosis, there is a relative lack of published studies on reasons for delays in teenagers. In a systematic literature search we found only one unpublished UK study on teenage cancer diagnostic delay, which concluded that delay was mainly attributable to the actions of healthcare professionals [24].

Yet, reasons for delay may vary by age. Indeed, this study of adolescents suggests that the most widely endorsed barriers to help-seeking were emotional barriers (‘worry about what the doctor might find’ [71.8%], ‘too embarrassed’ [55.6%], ‘too scared’ [54.4%], and ‘not feeling confident to talk about symptoms’ [53.3%]), whereas a UK benchmarking study found that the most widely endorsed barriers by adults were service barriers (‘difficulty making an appointment’ [40.7%] and ‘worried about wasting doctor’s time’ [38.1%]) [22]. Thus, future interventions to reduce patient time to presentation, diagnosis and cancer treatment should differ by age group. In particular, our study suggests that interventions to improve early diagnosis in teenagers should focus on addressing emotional barriers. Clearly more research on reasons for teenage cancer delay and interventions are required, which should be informed by theories which help us understand the interplay of psychosocial development and changes in cancer awareness.

The influence of social networks in promoting help-seeking are poorly understood and may vary by age. Recent research found that men who requested a PSA test (a form of help-seeking) were more likely to have friends with prostate cancer, suggesting that knowing someone with cancer facilitates help-seeking [25]. The impact on early diagnosis may extend beyond the individual with the symptom and prompt the presentation of friends and relatives to take action. This study adds to understandings of the influence of social networks by examining the influence of knowing someone with cancer on barriers to help-seeking among adolescents. Adolescents in this study who knew someone with cancer endorsed significantly more barriers to help-seeking than those who did not. The mechanisms through which knowing someone with cancer impacts perceived barriers to help-seeking are not known but are likely to be mediated by the closeness of their relationship to that person and outcome (e.g., whether that individual died from the disease). Further research with adolescents and the development of theoretical models to explain associations between social networks, emotions and health behaviours is required.

### Future research priorities

This study has identified three key priorities for future research. First, a nationally representative study should be conducted using a modified CAM validated with adolescents to benchmark adolescents’ cancer awareness across the UK. This would enable further comparison between adolescents’ and adults’ cancer awareness and cross-national comparison of adolescents’ cancer awareness. Second, further research is required into adolescents’ help-seeking behaviour and particularly how emotions, peer social networks and familial experience and relationships influence delay. This research would contribute to the development of clinically-relevant and age-appropriate theoretical models of adolescents’ delay. Third, there is a need to evaluate the effectiveness of existing interventions that seek to raise adolescents’ cancer awareness and address perceived barriers to help-seeking and assess their effects on family members, such as siblings and parents, through processes of diffusion. This would start to identify certain groups who may be more or less receptive to cancer messages to support the further development of individual- and group-level awareness-raising interventions. Advancing this research agenda along these three avenues would actively support current policy initiatives, such as NAEDI and DCE, that encourage early cancer diagnosis in adolescence and adulthood.

### Strengths and limitations

A strength of this study is the use of the CAM which meant that this study of adolescents used the same definitions, terms and measures of cancer awareness used in studies of adults. This consistency enables direct comparison to national baseline data and facilitates the construction of an evidence-base to support the development of interventions. Moreover, this study also provides an indication that the CAM is likely to be an acceptable tool to use with adolescents without considerable modification. However, further validation of the tool is advised before extensive use with adolescents. To our knowledge, this is the largest study of adolescent cancer awareness conducted in Britain, and among the largest internationally. The UK CAM benchmarking study included 170 individuals aged between 16 and 24 years old [2], and previous studies of adolescent cancer awareness had sample sizes ranging from 66 [6] to 274 [5]. Although one study included 4,002 adolescents these were of younger age (8 to 11 years old) [7].

The main limitation of this study is the use of non-probabilistic sampling which restricts the ability to make population inferences. However, we have no reason to believe that the adolescents in this study are systematically different from others in the UK. A further limitation of the study is the lack of analysis by socio-economic status (SES) as cancer awareness is known to vary by SES [2]. Future studies should therefore include individual-level measures of SES, such as free school meal entitlement or National Statistics Socio Economic Classification (NS-SEC) derived from parents’ occupation. Finally, it may be possible that parents and peers exert an influence on adolescents’ help-seeking behaviour. Thus, future research should develop and include measures of these influences.

## Conclusions

Overall, if the objectives of NAEDI and DCE are to be achieved then cancer awareness should be addressed in adolescence. There are certain groups of adolescents whose cancer awareness is poor and thus messages about cancer need to be targeted and tailored to particular groups of adolescents to prevent the development of health inequalities in adulthood. Research into adolescents’ cancer awareness is an emerging field which would benefit from application and development of theoretical frameworks to understand the patterns of cancer awareness and mechanisms that explain relationships between awareness and help-seeking. This will help to ensure a systematic and rigorous approach to the development of interventions to increase cancer awareness and help-seeking behaviour among adolescents which will contribute to their own early diagnosis, as well as potentially that of friends and relatives, and thereby survival throughout the lifecourse.

## Abbreviations

ANCOVA: Analysis of Covariance; CAM: Cancer Awareness Measure; DCE: Detect Cancer Early; GP: General Practitioner; NAEDI: National Awareness and Early Diagnosis Initiative; NS-SEC: National Statistics Socio-Economic Classification; PSA: Prostate Specific Antigen; SD: Standard Deviation; SES: Socio-Economic Status; TYA: Teenage and Young Adult.

## Availability of supporting data

Data collected for the purposes of this study are available through the UK Data Archive in accordance with the conditions of use of the CAM.

## Competing interests

The authors declare that they have no competing interests.

## Authors’ contributions

RGK developed database and managed data entry, designed and conducted data analysis and interpretation, drafted and revised the manuscript. LF conducted data interpretation, drafted and revised the manuscript. GH secured funding and ethics approval, managed data collection, conducted data interpretation, drafted and revised the manuscript. All authors read and approved the final manuscript.

## Pre-publication history

The pre-publication history for this paper can be accessed here:

http://www.biomedcentral.com/1471-2458/12/580/prepub
